# Improved UAV-to-Ground Multi-Target Tracking Algorithm Based on StrongSORT

**DOI:** 10.3390/s23229239

**Published:** 2023-11-17

**Authors:** Xinyu Cao, Zhuo Wang, Bowen Zheng, Yajie Tan

**Affiliations:** School of Computer and Control Engineering, Northeast Forestry University, Harbin 150006, China; c1404399208@gmail.com (X.C.); zbw609676671@163.com (B.Z.); tyj15294174958@163.com (Y.T.)

**Keywords:** unmanned aerial vehicle, small target detection, StrongSORT, OSNet

## Abstract

Unmanned aerial vehicles (UAV) are essential for aerial reconnaissance and monitoring. One of the greatest challenges facing UAVs is vision-based multi-target tracking. Multi-target tracking algorithms that depend on visual data are utilized in a variety of fields. In this study, we present a comprehensive framework for real-time tracking of ground robots in forest and grassland environments. This framework utilizes the YOLOv5n detection algorithm and a multi-target tracking algorithm for monitoring ground robot activities in real-time video streams. We optimized both detection and re-identification networks to enhance real-time target detection. The StrongSORT tracking algorithm was selected carefully to alleviate the loss of tracked objects due to factors like camera jitter, intersecting and overlapping targets, and smaller target sizes. The YOLOv5n algorithm was used to train the dataset, and the StrongSORT tracking algorithm incorporated the best-trained model weights. The algorithm’s performance has greatly improved, as demonstrated by experimental results. The number of ID switches (IDSW) has decreased by sixfold, IDF1 has increased by 7.93%, and false positives (FP) have decreased by 30.28%. Additionally, the tracking speed has reached 38 frames per second. These findings validate our algorithm’s ability to fulfill real-time tracking requisites on UAV platforms, delivering dependable resolutions for dynamic multi-target tracking on land.

## 1. Introduction

With the continual advancements in autonomous intelligence, unmanned aerial vehicles (UAV) have become integral to civil applications such as aerial photography and agricultural exploration, as well as military operations, including reconnaissance, monitoring, and pursuit of criminal activity. The development of dynamic multi-target tracking technology of UAVs represents a significant challenge that requires immediate attention within the UAV field. For the dynamic task of multi-target tracking, the varied motion patterns of the targets pose significant challenges to the UAV system. Vision-based multi-tracking technology, known for its real-time performance, adaptability, and flexibility, has emerged as a crucial instrument to overcome this difficulty.

Dynamic multi-target recognition and tracking tasks have shown remarkable advancements in the areas of pedestrian detection [1], vehicle detection [2], and medical analysis [3]. Nevertheless, the direct application of such algorithms to UAVs is limited due to the different viewpoint of UAVs, which differs from the traditional perspective. Traditional target detection methods primarily depend on manual feature extraction, including VJ [4], HOG [5], and DPM [6]. However, these methods are more challenging when confronted with dynamic environments, changing backgrounds, and occlusion situations. In recent years, Convolutional Neural Networks (CNNs) have made significant progress in target detection, including Faster R-CNN [7], FPN [8], YOLO [9], and SSD [10]. Nonetheless, two-stage algorithms like Faster R-CNN, although highly accurate, are slow and difficult to further improve compared to one-stage algorithms.

Compared to research in target recognition and other areas, the field of object tracking began relatively late. It can be generally classified into two types: multi-object tracking based on target features and multi-target tracking with trajectory prediction. Among them, multi-target tracking based on target features is to track the target by extracting features. Representative algorithms such as OSIM [11], MAC [12], and Pas tracker [13] have demonstrated strong performance in scenarios with short-term occlusion. However, these algorithms face challenges in scenarios with significant appearance changes. The second category of algorithms uses target trajectory prediction to achieve multi-object tracking, using a Bayesian filter framework to capture dynamic object behavior and predict and update trajectories. Common methods for predicting target trajectories include Kalman filter and particle filtering, employing representative algorithms such as SORT [14], QEPF [15], ByteTrack [16], and OCSORT [17]. These methods can more accurately project trajectories in instances of long-term occlusion when compared to feature-based modeling algorithms. Nevertheless, traditional methods like the Kalman filter are not ideal for unstable shooting conditions or nonlinear motion [18] on drone platforms. The integration of target trajectory prediction and the re-identification networks through deep learning has emerged as an effective technique in addressing long-term tracking issues. Noteworthy algorithms that employ this approach include DeepSORT [19] and StrongSORT [20]. However, users may face challenges achieving real-time tracking with these methods because of their large models and longer detection times.

**Figure 1:**
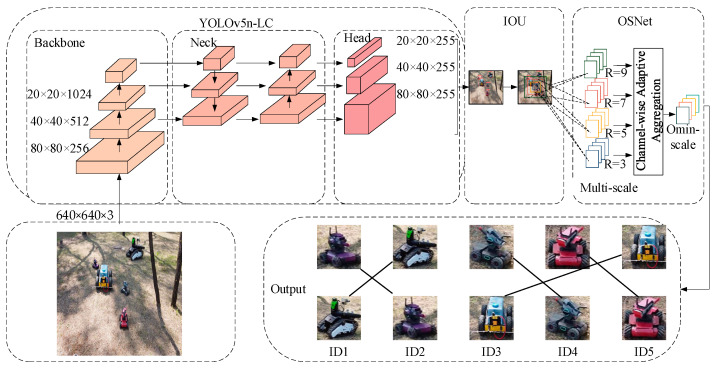
Successful tracking process: the input image is initially fed into the YOLOv5-LC to extract detected targets. Subsequently, matching is performed based on intersection over union (IOU), and when the same target is matched for three or more frames, it is considered successfully tracked. After successful tracking, OSNet is employed for feature extraction, facilitating direct target tracking.

In the realm of real-time multi-object tracking using UAV, especially in intricate settings such as forests and grasslands, we suggest a method called YL-SS to tackle prevailing difficulties. An overview of the tracking process is shown in Figure 1. The acronym “YL-SS” embodies its fundamental features, with “Y” denoting the use of the single-stage YOLOv5n [21] strategy for boosting tracking speed and adaptability. “L” denotes the use of the lightweight LCNet [22] as the backbone network, which improves small target detection in complex scenarios while emphasizing real-time advantages. “S” indicates inspiration drawn from the StrongSORT algorithm, which incorporates ECC (Enhanced Correlation Coefficient) camera motion compensation [23] and NSA Kalman (Noise Scale Adaptive Kalman algorithm) [24] to ensure high stability and robustness on UAV platforms. Additionally, the letter “S” signifies the implementation of the OSNet [25] as the model for the re-identification of targets to increase their adaptability to appearance changes. The accurate modeling of complex scenarios, including occlusion, variations in appearance, and interactions with multiple targets, is emphasized to tackle these challenges. These innovative strategies and technologies collectively comprise the YL-SS algorithm, which aims to enhance the performance and resilience of real-time multi-object tracking in dynamic scenarios. The success tracking process is shown in Figure 1. The primary components of this paper include:

(1) To reduce the parametric number of the model and improve the detection speed of the model deployed in the devices, The LCNet was used as the backbone of YOLOv5n for extracting features, and the depthwise separable convolution was used to substitute the normal convolution in the neck and head parts. Additionally, the C3Ghost [26] module was introduced to replace the original C3 module.
(2) The Focal–EIOU loss [27] was adopted as a replacement for the original CIoU loss, which not only focuses on high-quality anchor boxes but also accurately measures the overlap, center points, and side lengths of bounding boxes. These improvements allow for faster model convergence and more accurate regression results.
(3) Our research focuses on tracking robots, specifically specialized robots, in forest and grassland environments. To evaluate the performance of our algorithm in these specific application areas, we created a specialized dataset that accurately represents the challenges and environments encountered in these situations.
(4) To address the challenges of efficient multi-object tracking in diverse scenarios, we have adopted OSNet, a lightweight and efficient network architecture with superior object re-identification capabilities, which enables our system to perform robustly even in complex environments like forested areas.

The following sections provide an organized presentation of the paper’s content. Section 2 presents a detailed description of the YOLOv5n algorithm and the improved YOLOv5n-LC algorithm. Section 3 focuses on the StrongSORT optimization scheme and its detailed explanation. In Section 4, we introduce and analyze the evaluation metrics used in the paper and include experimental results. In Section 5, we provide a further discussion of the experimental results. Finally, in Section 6, we summarize the paper’s findings and suggest future research areas.

## 2. YOLOv5n-LC

### 2.1. YOLOV5n

In the context of dynamic tasks involving detecting and tracking multiple targets with UAVs, the selection of an appropriate detector model is a crucial step. Training results of the detector model have a direct effect on the effectiveness of target trajectory tracking. Moreover, the detection speed and accuracy of the target detector are both strongly linked to real-time target tracking performance. The YOLOv5n model is the chosen detector model for this research. YOLOv5 offers several versions, one of which is YOLOv5n, which is highly regarded. The reason for selecting YOLOv5n is due to its smaller size, lower computational requirements, and faster detection speed. These features make it particularly well-suited to meet the demands of real-time target tracking.

**Figure 2:**
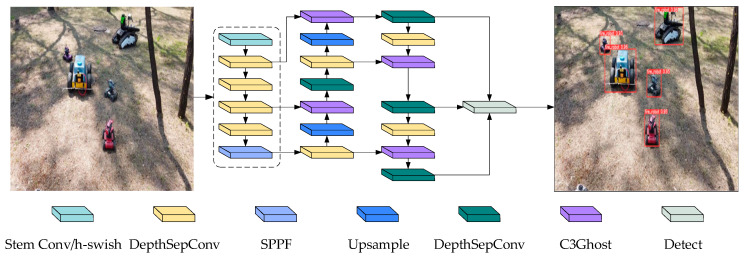
The YOLOv5-LC network model, as shown in the figure, incorporates significant improvements. These enhancements include replacing CSPDarknet53 with LCNet, using depthwise separable convolutions instead of regular convolutions, and employing C3Ghost to replace C3. These key improvements have resulted in reduced network complexity and an increased focus on feature information.

The architecture of the YOLOv5n network comprises four primary components: input, backbone, neck, and head. In the input section, the image size is adjusted to 640 × 640 × 3 and augmented with the Mosaic method to enhance the data. Then, the backbone is utilized to extract features from the processed images. After feature extraction, the neck module fuses and processes the features to create large, medium, and small features of different scales, which are then fed into the detection head for object detection. Our modifications mainly target the backbone, neck, and loss function components. Figure 2 illustrates the modified network model.

### 2.2. Backbone: LCNet

LCNet utilizes DepthSepConv as the core module [28] and has undergone several optimizations. The fundamental structural design of LCNet employs branchless DepthSepConv, which significantly accelerates the inference process. Additionally, the silu activation function [29] in the backbone has been replaced with the superior H-Swish [30], which effectively reduces the calculation overhead. Additionally, the network has been enhanced by inserting an SE channel attention mechanism module [31] at the end to increase the emphasis on valuable feature information. Furthermore, the use of 5 × 5 convolution kernels has replaced deep 3 × 3 convolutions, resulting in a larger receptive field. 

### 2.3. Neck

The neck and head of YOLOv5n contain multiple 3 × 3 convolutional structures, which significantly increases the network parameters and computation, thereby impacting the detection speed. To mitigate the network’s complexity, this paper utilizes depthwise separable convolution [32] as a substitute for the conventional 3 × 3 convolution. This substitution effectively reduces the number of parameters.

**Figure 3:**
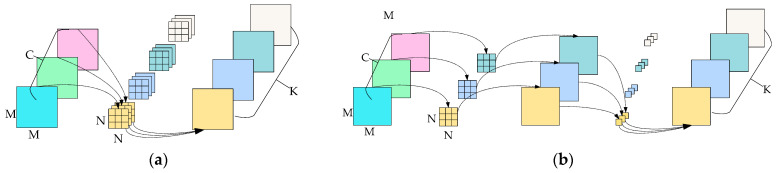
Comparison of general convolution and depthwise separable convolution. The proportion of the computation of depthwise separable convolution to that of general convolution is calculated as follows, given an input size of M × M × C, a convolutional kernel size of N × N, and an output channel size of K: $\frac{M\times M\times C\times N\times N+K\times C\times M\times M}{M\times M\times C\times K\times N\times N}=\frac{1}{K}+\frac{1}{N^{2}}$. In general, N = 3 and K > 1, so that depthwise separable convolution requires relatively little computation. (**a**) Standard convolution; (**b**) depthwise separable convolution.

Figure 3 illustrates the process of extracting features from the general convolution and the depthwise separable convolution. The standard convolution, depicted in Figure 3a, operates on images with three input channels and applies the convolution process on each input channel in a single step. In contrast, the depthwise separable convolution, shown in Figure 3b, consists of two stages: depthwise convolution and point convolution. In the depthwise convolution process, a separate filter is applied to each channel of the input to perform the convolution operation. On the other hand, in the point convolution, the dimensionality is increased by using a convolution kernel with a size of 1 × 1.

**Figure 4:**
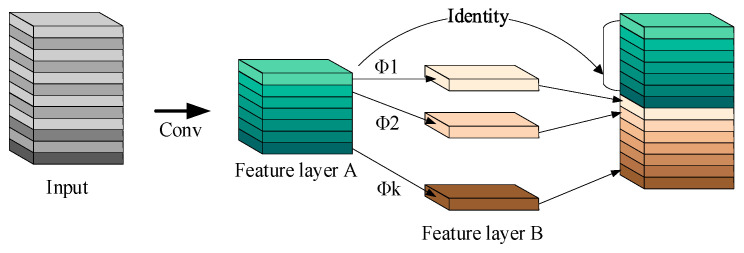
The convolution process of the Ghost module.

We also included the C3Ghost module [26], which includes GhostConv, to replace the original C3 module. The GhostConv operation, as shown in Figure 4, generates a subset of feature maps using a smaller number of convolutional kernels. Then, channel-wise convolution is applied to these feature maps to produce additional feature maps. Finally, the two sets of feature maps are concatenated to produce the GhostNet feature maps. This modification further reduces computational complexity and improves efficiency.

### 2.4. Loss Function

The CIOU loss function [33] in YOLOv5n effectively handles several issues during bounding box regression, which results in the quicker convergence of predicted boxes. Nonetheless, it can create a problem where the predicted boxes’ width and height do not converge at a fixed ratio. To resolve these issues, the EIOU loss [27] and its computational form, demonstrated in Equation (1), are taken into account.

(1) $$L_{EIOU}=L_{IOU}+L_{dis}+L_{asp}=1-IOU+\frac{\rho^{2}\left(b,b^{gt}\right)}{C^{2}}+\frac{\rho^{2}\left(w,w^{gt}\right)}{C_{w}^{2}}+\frac{\rho^{2}\left(h,h^{gt}\right)}{C_{h}^{2}}$$

In the context of predicting bounding box regression, training samples are essential for convergence. Therefore, the Focal–EIOU [27] loss is employed, under the framework of the EIOU loss, to replace the initial CIOU bounding box regression loss in YOLOv5. In addition, a suppression factor γ is introduced, and its computational formula is defined in Equation (2).

(2) $$L_{Focal-EIOU}=IOU^{\gamma }L_{EIOU}$$

This method not only focuses on high-quality anchor boxes but also accurately measures the overlap, center points, and side lengths of bounding boxes. These enhancements facilitate swifter model convergence and more precise regression outcomes. Additionally, the approach tackles the challenge of bounding box disparity and demonstrates robustness, particularly in small dataset-oriented situations.

## 3. Optimization of Strong SORT

**Figure 5:**
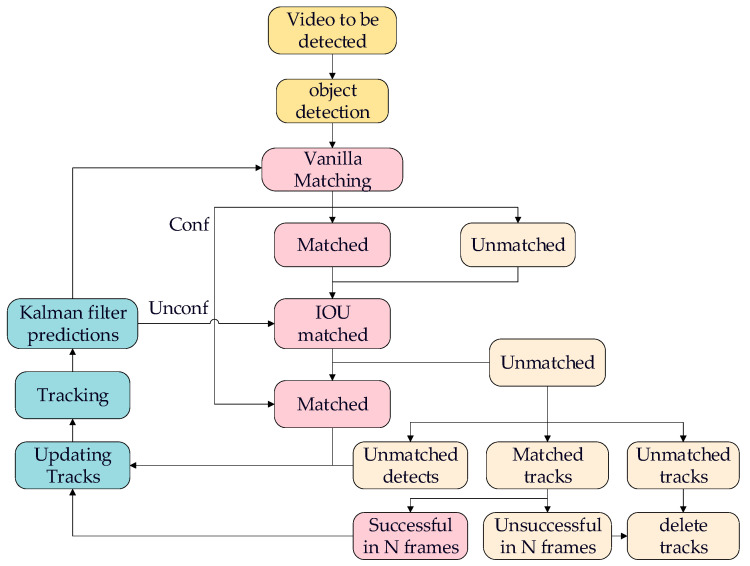
YL-SS algorithmic framework, N is set to 100.

The entire algorithm framework is illustrated in Figure 1. Firstly, the video to be detected is passed through the object detection module for target detection. We use YOLOv5n-LC as the detector, which outputs information about the detected target boxes. Next, the feature extraction network is constructed using the full-scale network (OSNet) within the tracking module to extract the appearance features of the targets. Simultaneously, the NSA Kalman method within the tracking module is employed to update and predict the position information of the targets. Finally, through vanilla and IOU matching methods, the results from the object detector are associated with the predictions from the tracking module. Based on the vanilla matching results, if the vanilla is successful, the next frame can be updated and predicted directly. In cases where the matched target disappears but reappears in N frames, the tracking can be confirmed and the next frame can be predicted. In the case of a new target appearing without a tracking frame, the IOU matching can be used. If three consecutive matches are successful, it will be considered a confirmed state. The algorithmic framework is shown in Figure 5.

### 3.1. NSA Kalman

The traditional Kalman filter [34] uses a recursive approach to estimate past, current, and future states of detection boxes. They are a practical, efficient, and widely applied method for state estimation in solving engineering problems. However, in dynamic multi-object tracking scenarios with UAVs, challenges such as environmental changes, sensor drift, and varying noise levels arise. Applying a uniform measurement noise scale to all detection boxes without considering detection quality cannot yield more precise motion states. Thus, in the camera module, we introduce camera motion compensation using the enhanced correlation coefficient maximization (ECC) [23], as presented in Equation (3).

(3) $${E_{ECC}\left(p\right)=\|\frac{{\bar{i}}_{r}}{\left\|{\bar{i}}_{r}\right\|}-\frac{{\bar{i}}_{w}\left(p\right)}{\left\|{\bar{i}}_{w}\left(p\right)\right\|}\|}^{2}$$

In the following, ||·||denotes the Euclidean norm, *p* is the deformation parameter, and ${\bar{i}}_{r}$ and ${\bar{i}}_{w}\left(p\right)$ are zero-mean versions of the reference image ir and the deformed image *i_w_*(*p*). To address the image alignment issue, an iterative algorithm, either forward additive or inverse compositional, is used to minimize *E_ECC_* (*p*). The ECC algorithm [35] is an effective solution for reducing motion noise for UAVs. Its efficiency makes it a reliable tool for this purpose.

Subsequently, Du, Y proposed the NSA Kalman [24], which addresses the issue of detection quality by adaptively adjusting the noise scale based on the quality of the target detection boxes. The adaptive computation of the noise covariance ${\tilde{R}}_{k}$ in Equation (4) is determined by the following formula:

(4) $${\tilde{R}}_{k}=\left(1-C_{k}\right)R_{k}$$

where $R_{k}$ represents the measurement noise covariance constant and $C_{k}$ denotes the detection confidence score for state k. A lower noise level leads to a higher confidence score $C_{k}$ for the detection, resulting in a decreased value for ${\tilde{R}}_{k}$. A lower value for ${\tilde{R}}_{k}$ indicates a higher weight for detection in the state update step and vice versa. This approach helps improve the accuracy of the updated state.

### 3.2. Target Re-Identification

ResNet50 [36], as a versatile feature extractor, has demonstrated outstanding performance in the ImageNet large-scale image classification competition and has been widely used in various image processing tasks: [20,37,38], among others. However, its large model size and number of parameters can lead to increased time and computational resource requirements for image processing, making it less suitable for resource-constrained UAV platforms. In contrast, the OSNet network is specifically designed for tasks such as target re-identification and has shown excellent performance in these specific tasks, along with higher efficiency and adaptability. Therefore, we decided to use OSNet to replace ResNet50 in StrongSORT and trained on it using the NEF dataset.

Furthermore, we adopted the feature storage and update strategy proposed in [39], which takes into account both multi-frame information and inter-frame variation information. This strategy is better suited to achieve more accurate associations between detection and tracking in complex scenarios. The algorithm uses the Exponential Moving Average (EMA) to update the appearance state of the i-th track at the t-th frame in the feature extraction branch. The Equation (5) is as follows.

(5) $$e_{i}^{t}=\alpha e_{i}^{t-1}+\left(1-\alpha \right)f_{i}^{t}$$

where $f_{i}^{t-1}$ is the appearance embedding of the currently matched detection, and we set α (motion vector) = 0.9. The EMA update strategy not only improves the quality of matching but also reduces time consumption.

## 4. Experiment and Analysis

### 4.1. Construction of Dataset

We created our own target detection and target tracking datasets. For the detection part, we collected data using two different UAVs, called the NEF dataset. We collected data in two different scenarios: an open grassy area without obstacles and a forested area with obstacles, such as trees. In addition, for the ground robots, we used different sizes of robots for different scales. We used a DJI PHANTOM 3 STANDARD at 5 m and 15 m, two different heights, to record situations such as overlapping in the motion of dynamic multi-targets on the ground in the stationary state of the UAV, as well as the shooting of dynamic multi-targets on the ground by the UAV at a constant speed of 2 m/s, and the occurrence of camera shake when the UAV suddenly stops, respectively. In total, four video clips were collected, from which 2390 frames were extracted. The image size is 1920 × 1280 pixels. The dataset is divided into training and validation sets in the ratio of 80:20, with the training set containing 1904 frames and the validation set containing 332 frames.

In the target tracking section, we cropped the different targets in a single image and resized them to a uniform format of 128 × 64 pixels. Then, we organized them into their respective folders. Regarding the annotation of the metrics for evaluating the target tracking results, we annotated a total of 2012 images for evaluating the tracking results.

### 4.2. Evaluation Metrics

In terms of detection, the evaluation metrics used in this experiment include accuracy (P), recall (R), F1 score (F1), mean average precision (mAP), frames per second (FPS), and model size. Accuracy refers to the correctness of predictions among all samples predicted as positive. Recall measures the correctness of predictions among all truly positive samples. The F1 score is the harmonic mean of precision and recall. Precision, recall, and F1 score can be calculated using the following formulas:

(6) $$\mathrm{Precision}=\frac{\mathrm{TP}}{\mathrm{TP}+\mathrm{FP}}$$

(7) $$\mathrm{Recall}=\frac{\mathrm{TP}}{\mathrm{TP}+\mathrm{FN}}$$

where TP represents correctly detected targets, FN is for missed targets, and FP represents falsely detected targets. mAP refers to the mean average precision across all detected target classes, which can be calculated using the following formulas:

(8) $$F1=\frac{2\times \mathrm{Percision}\times \mathrm{Recall}}{\mathrm{Percision}+\mathrm{Recall}}$$

(9) $$AP=\int_{0}^{1}P\left(R\right)dR$$

In terms of tracking, the evaluation metrics mainly contain the following parameters: IDSw, MOTA, IDP, IDR, IDF1. The correlation formula is

(10) $$\mathrm{MOTA}=1-\frac{\mathrm{FN}+\mathrm{FP}+\mathrm{IDSw}}{\mathrm{GT}}$$

(11) $$\mathrm{IDP}=\frac{\mathrm{IDTP}}{\mathrm{IDTP}+\mathrm{IDFP}}$$

(12) $$\mathrm{IDR}=\frac{\mathrm{IDTP}}{\mathrm{IDTP}+\mathrm{IDFN}}$$

(13) $$\mathrm{IDF}1=\frac{2}{\frac{1}{\mathrm{IDP}}+\frac{1}{\mathrm{IDR}}}=\frac{2\mathrm{IDTP}}{2\mathrm{IDTP}+\mathrm{IDFP}+\mathrm{IDFN}}$$

where TP represents the total number of false positives, FP represents the total number of falsely detected targets, FN represents the total number of false negatives (missed targets), IDSw is the Number of Identity Switches, MOTA represents Multi-Object Tracking Accuracy, GT is the number of Ground Truth objects. IDP is identification precision, IDR is identification recall, IDTP indicates that the identification is the same before and after each frame, IDFP represents the change in frame identification before and after each, IDFN represents a post-frame not detected identification, IDF1 represents the ratio of correctly identified detections over the average number of ground-truth and computed detections.

The experimental setup included a Windows 11 operating system, an Intel Core i7-12700H CPU, an Nvidia GeForce RTX 3060 graphics card, 16 GB of RAM, and software tools such as CUDA 11.1. The deep learning acceleration library cuDNN 8.8.0. PyTorch 1.9.0 was used as the framework, and training and validation of the target detection and tracking models were performed within the PyCharm integrated development environment (IDE).

### 4.3. Analyzes of Results

#### 4.3.1. Performance Assessment Results

**Figure 6:**
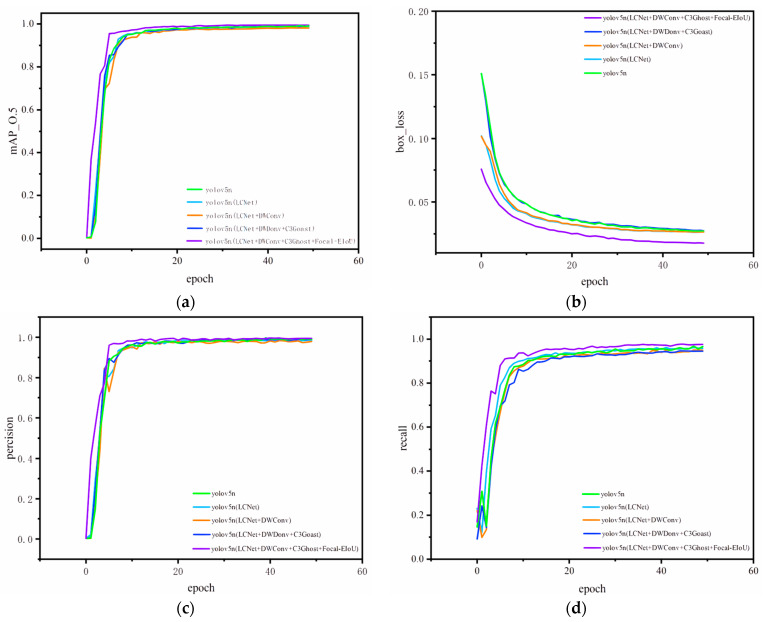
YOLOv5n-LC was added to the ablation experiments of different modules, respectively, and the results of 50 rounds of training on the NEF dataset are compared. (**a**) mAP0.5 curve comparison; (**b**) loss curve comparison; (**c**) precision curve comparison; (**d**) recall curve comparison.

The study displays the visualization of YOLOv5n-LC training on the NEF dataset for 50 epochs in Figure 6. Minimal fluctuations in the precision and recall curves can be observed, along with gradual convergence of the bounding box regression loss on both the training and validation sets with very little oscillation. These findings suggest that the model did not experience overfitting or underfitting during the training process. The metric/mean average precision at a 0.5 intersection over union steadily increases and stabilizes, indicating that the optimized model has exceptional learning capabilities.

#### 4.3.2. Ablation Experiments

In order to maintain a fair comparison, all training parameters were kept constant except for the improvements made during the experimental process. The dataset used comprised images with a resolution of 640 × 640 as input. The training results are presented in Table 1.

After replacing YOLOv5n’s feature extraction structure with LCNet structure, the model’s detection time, number of parameters, computational complexity, and model volume all decreased. Specifically, the detection time decreased by 0.4 ms, the number of parameters decreased by 7.74 × 10^5^, the computational complexity decreased by 2.2 GFLOPs, and the model volume decreased by 1.49 MB. Additionally, the Conv modules and C3 modules in the feature fusion section of YOLOv5n were completely replaced with depth-wise separable convolution modules and C3Ghost modules. The Table 1 data show that this process has further reduced the weight of YOLOv5n.

The results show that the final improved YOLOv5n-LC improves various key metrics compared to the fusion of YOLOv5n and LCNet, including a reduction in the number of parameters by 4.61 × 10^5^, a reduction in computational complexity by 0.8 GFLOPs, a reduction in the model volume by 0.86 MB, and a reduction in the detection time by 0.2 ms.

This improvement is primarily attributed to the integration of the Focal–EIOU loss function, which balances accuracy and convergence in object detection. The enhanced network leverages depth-wise separable convolution, SE attention mechanism modules, and C3Ghost modules, replacing complex convolution operations with straightforward linear operations. These changes do not compromise the mean average precision (mAP), and they significantly enhance the operational speed of the detection end.

In summary, the modified YOLOv5n-LCNet structure optimally balances various performance metrics while maintaining the same high level of mAP in object detection.

#### 4.3.3. Comparison Experiment

In real-time dynamic multi-target tracking based on TBD, the overall tracking rate is directly affected by the detection rate at the detection end. Five models are compared: YOLOv3-tiny [40], YOLOv5s [21], YOLOv5n, YOLOv7-tiny [9], and the improved YOLOv5-LC, each trained with pre-trained weights. Under the same circumstances, the NEF dataset that was created by us was used for training purposes. The performance of the detectors was evaluated based on mAP, computation time, number of parameters, detection time, and model volume. The results of the experiments are presented in Table 2.

It can be seen that our improved YOLOv5n-LC reaches 3.6 ms, 1.1 GFLOPs, and 1.29 MB in terms of detection rate, computation, and model size on the validation dataset, followed by YOLOV5 as well as YOLOv3-tiny target detectors. The slowest detection rate is achieved by YOLOv7-tiny, with a detection rate of only 8.8 ms, compared to YOLOv7-tiny with the highest detection accuracy. Compared to YOLOv7-tiny, YOLOv5n-LC exhibits improvements in detection rate, FLOPs, and model size by 59.09%, 91.60%, and 79.19%, respectively.

#### 4.3.4. Comparison Experiment

In the context of ground-based dynamic multi-object tracking, we compared DeepSORT, ByteTrack, StrongSORT, and the improved YL-SS algorithm. Each model was trained with pre-trained weights under the same conditions, using our custom NEF dataset for training. We evaluated the trackers based on IDF1, FP (false positives), IDSW (ID switches), and detection time, among other metrics. The experimental results are shown in Table 3.

The presented results in Table 3 indicate that the ByteTrack algorithm achieves the fastest detection speed when compared to the YL-SS algorithm. However, there is a noteworthy increase in ID switches by 233%, a total false positive increase of 42.17%, and a decrease in ID tracking accuracy and recall by 6.11%. In contrast, the YL-SS algorithm maintains real-time capability while considerably decreasing important metrics like ID switches and ID tracking accuracy and recall when compared to DeepSORT. Consequently, it stands as a robust choice for this task. Furthermore, despite its minor drop in accuracy when weighed against the precision-based StrongSORT algorithm, the YL-SS algorithm results in a 37% increase in detection speed.

In conclusion, the YL-SS algorithm is well suited for the task at hand as it achieves an effective balance between real-time processing and robust performance compared to the Strong SORT algorithm with OSNet.

## 5. Discussion

**Figure 7:**
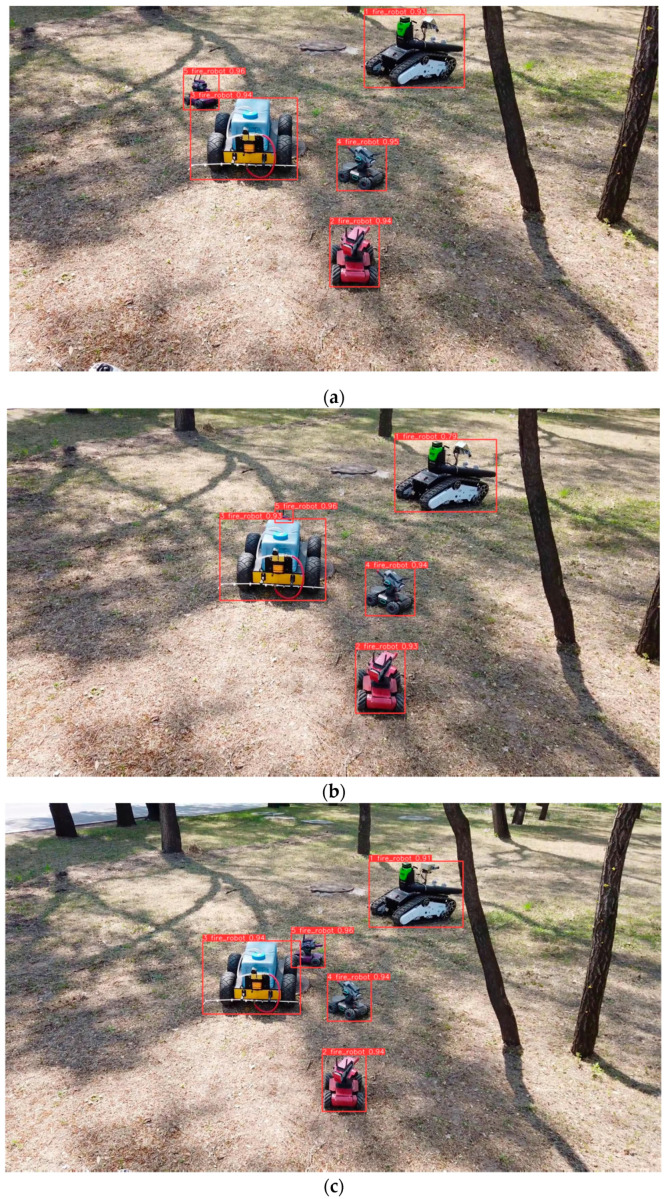
Low-altitude scene images (**a**) frame 50; (**b**) frame 66; (**c**) frame 132. From (**a**–**c**), target #5 is still tracked accurately when it is in occlusion from frame 60 to 132.

**Figure 8:**
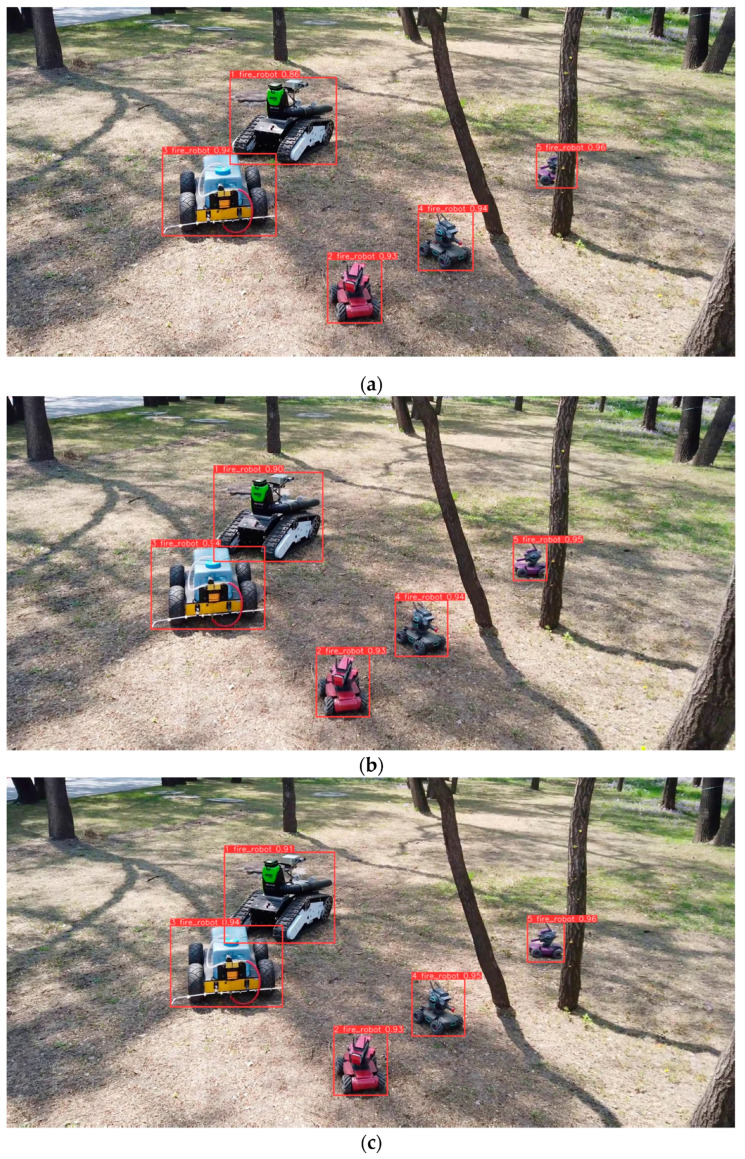
Low-altitude scene images (**a**) frame 397; (**b**) frame 402; (**c**) frame 407. From (**a**–**c**), there is camera shake from frame 402 to 407, still tracking stably.

**Figure 9:**
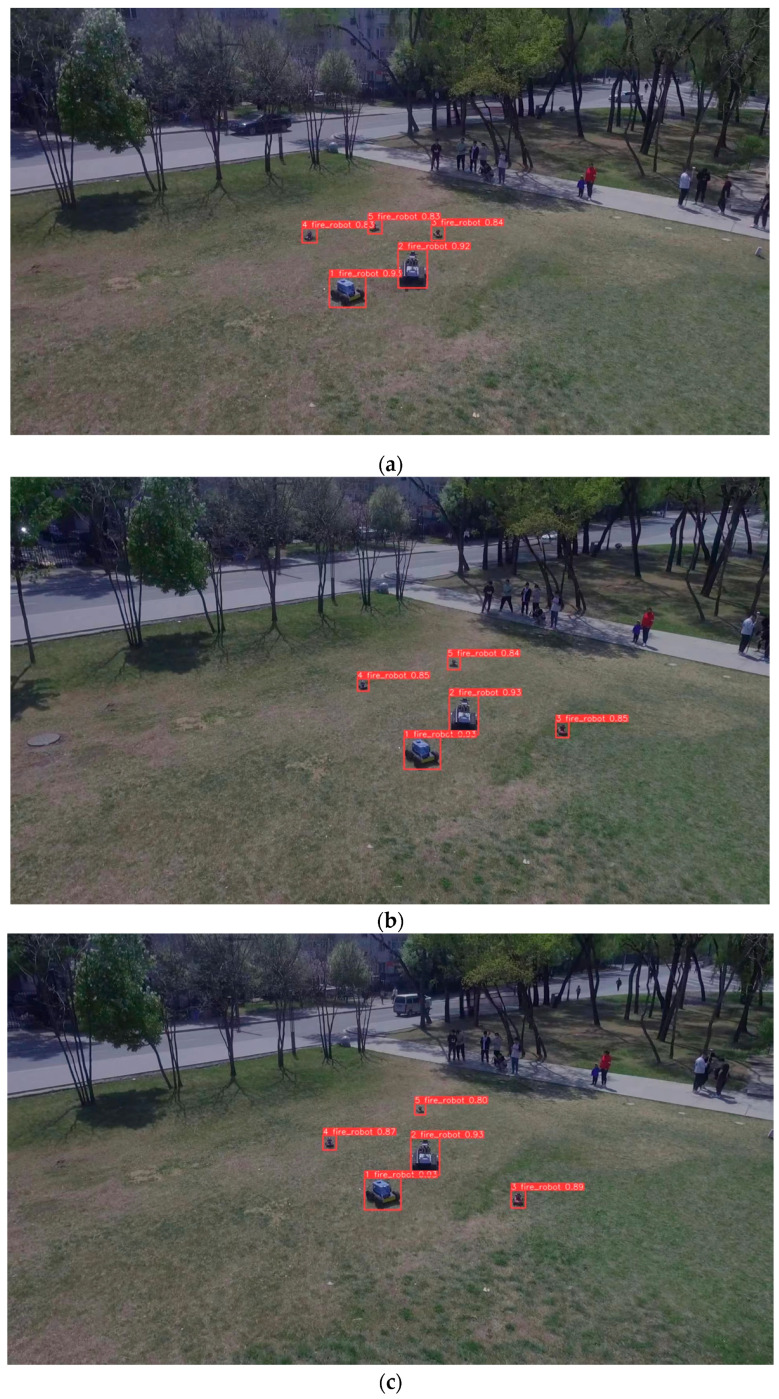
High-altitude scene images (**a**) frame 3; (**b**) frame 111; (**c**) frame 233. From (**a**–**c**), frame 3 to 233 long tracking can still be maintained for small targets.

In our real-time on-ground dynamic multi-object tracking system, we address challenges related to target detection, target re-identification, and Kalman filtering. Our focus is on achieving real-time performance and handling transient occlusions. In our experiments, the YL-SS algorithm employs a lightweight detector and a re-identification network specifically designed to address occlusion issues. This approach ensures real-time capability and demonstrates excellent performance in scenarios involving camera jitter, overlapping and intersecting targets, and small-sized objects, as shown in Figure 7, Figure 8 and Figure 9. On the other hand, the ByteTrack algorithm has the fastest detection rate but does not employ a target re-recognition network. It has too many target switches when facing camera jitter and overlapping targets. In addition, StrongSORT and DeepSORT algorithms use a generalized target re-identification network, which has good performance in the face of the occlusion problem, but their long detection time prevents real-time tracking.

The experimental results validate the accuracy of the YL-SS algorithm. By replacing the backbone network with LCNet and modifying the neck network to utilize DSConv and C3Ghost instead of Conv and C3, the improved network exhibits significant enhancements in detection speed, computational complexity, and model size. The substantial improvement in detection speed during multi-object tracking confirms the effectiveness of the optimized detection component. Additionally, the adoption of OSNet, ECC camera motion compensation, and NSA Kalman demonstrates superior performance compared to other tracking algorithms. On the other hand, there are cases of target switches observed during prolonged occlusions. This may be attributed to potential sensor noise that can result in the inaccurate measurements of target positions or features.

## 6. Conclusions

We present an enhanced multi-target tracking algorithm for unmanned aerial vehicles (UAV) designed to handle the challenges of real-time target detection with small sizes, varying scales, and background interference. To achieve improved detection performance, we optimized the feature extraction module using LCNet and depthwise separable convolution and employed C3Ghost to enhance detection quality. Additionally, we introduced the Focal–EIOU loss function to heighten target localization accuracy. For multi-target tracking, we use an all-scale network (OSNet) instead of ResNet to improve our ability to tackle the multi-target tracking task. Our experiments demonstrate that the YL-SS algorithm we implement has a faster processing speed and resolves situations where target ID loss or switching occurs due to long-term occlusion and significant changes in motion scales. Additionally, we have achieved good results in speed detection and tracking. Moving forward, we will examine how to apply this research to embedded terminal applications.

## Figures and Tables

**Table 1:** Results of ablation experiments (optimal performance is bold).

Models	mAP/% ↑	Time (ms) ↓	Param/10^6^ ↓	FLOPs (G) ↓	Model Size (MB) ↓
YOLOv5n	99.10	4.4	1.76	4.1	3.64
+LCNet	98.42	4.0	0.986	1.9	2.15
+DSConv	98.08	3.9	0.763	1.6	1.73
+C3Ghost	99.06	3.8	0.525	1.1	1.29
+Focal–EIoU Loss	**99.44**	**3.6**	**0.525**	**1.1**	**1.29**

↓ indicates better performance (lower is better). ↑ indicates better performance (higher is better).

**Table 2:** Comparison results of different models (optimal performance is bold).

Models	mAP/% ↑	Time (ms) ↓	Param/10^6^ ↓	FLOPs (G) ↓	Model Size (MB) ↓
YOLOv3-tiny	99.40	5.4	8.7	12.9	16.6
YOLOv7-tiny	**99.60**	8.8	6.03	13.1	6.2
YOLOv5s	99.50	5.9	7.0	15.8	13.6
YOLOv5n	99.10	4.4	1.7	4.1	3.64
YOLOv5n-LC	99.44	**3.6**	**0.53**	**1.1**	**1.29**

↓ indicates better performance (lower is better). ↑ indicates better performance (higher is better).

**Table 3:** Comparison results of different models (optimal performance is bold).

Models	IDF1 ↑	IDP ↑	IDR ↑	FP ↓	IDSW ↑	MOTA ↑	Time (ms) ↓
Deep SORT	59.8	59.8	59.8	485	40	88.9	31.8
ByteTrack	81.4	81.3	81.5	445	20	90.2	**11.1**
Strong SORT	80.3	80.3	80.3	449	8	90.2	41.7
YL-SS (Improved)	**86.7**	**88.0**	**85.4**	**313**	**6**	**90.4**	26.2

↓ indicates better performance (lower is better). ↑ indicates better performance (higher is better).

## Data Availability

The NEF dataset provided in this study can be requested via email: c1404399208@gmail.com.
